# Metabolic Adaptations in an Endocrine-Related Breast Cancer Mouse Model Unveil Potential Markers of Tumor Response to Hormonal Therapy

**DOI:** 10.3389/fonc.2022.786931

**Published:** 2022-03-01

**Authors:** Rita Araújo, Victoria Fabris, Caroline A. Lamb, Claudia Lanari, Luisa A. Helguero, Ana M. Gil

**Affiliations:** ^1^ Department of Chemistry and CICECO - Aveiro Institute of Materials (CICECO/UA), University of Aveiro, Campus Universitário de Santiago, Aveiro, Portugal; ^2^ Instituto de Biología y Medicina Experimental (IByME), Buenos Aires, Argentina; ^3^ Institute of Biomedicine (iBIMED), Department of Medical Sciences, Universidade de Aveiro, Aveiro, Portugal

**Keywords:** endocrine-related breast cancer, murine model, metabolomics, metabonomics, therapy resistance, biomarkers

## Abstract

Breast cancer (BC) is the most common type of cancer in women and, in most cases, it is hormone-dependent (HD), thus relying on ovarian hormone activation of intracellular receptors to stimulate tumor growth. Endocrine therapy (ET) aimed at preventing hormone receptor activation is the primary treatment strategy, however, about half of the patients, develop resistance in time. This involves the development of hormone independent tumors that initially are ET-responsive (HI), which may subsequently become resistant (HIR). The mechanisms that promote the conversion of HI to HIR tumors are varied and not completely understood. The aim of this work was to characterize the metabolic adaptations accompanying this conversion through the analysis of the polar metabolomes of tumor tissue and non-compromised mammary gland from mice implanted subcutaneously with HD, HI and HIR tumors from a medroxyprogesterone acetate (MPA)-induced BC mouse model. This was carried out by nuclear magnetic resonance (NMR) spectroscopy of tissue polar extracts and data mining through multivariate and univariate statistical analysis. Initial results unveiled marked changes between global tumor profiles and non-compromised mammary gland tissues, as expected. More importantly, specific metabolic signatures were found to accompany progression from HD, through HI and to HIR tumors, impacting on amino acids, nucleotides, membrane percursors and metabolites related to oxidative stress protection mechanisms. For each transition, sets of polar metabolites are advanced as potential markers of progression, including acquisition of resistance to ET. Putative biochemical interpretation of such signatures are proposed and discussed.

## Introduction

Breast cancer (BC) accounts for nearly 25% of diagnosed cancer cases in women worldwide, causing the highest number of deaths by cancer in this population. About 70% of all BC cases express estrogen receptor α (ERα) and progesterone receptor (PR), being referred to as hormone receptor (HR)-positive BC (1). Since HR-positive breast tumors depend on activation of ERα to sustain growth, most endocrine therapies target the ER signalling pathway (2). Current therapeutic strategies include blocking ERα transcriptional activity with tamoxifen or fulvestrant, and blocking estrogen conversion in peripheral tissues with aromatase inhibitors (3). PR can also sustain tumor growth and, thus, endocrine therapies targeting PR are also beginning to be used in selected cases (4–6). The most common progestins used for BC treatment in advanced stages are megestrol acetate and medroxyprogesterone acetate (MPA) (6), while antiprogestins, including RU486 (mifepristone) and onapristone, have been used to treat BC patients for whom other treatments have failed (6, 7). Although the vast majority of BC patients initially respond to endocrine therapy (ET), about 30-50% eventually relapse (acquired resistance), while *ca.* 20-30% never respond (*de novo* resistance) (8). Resistance to ET results from a variety of cellular changes that converge in metabolic adaptations, the overexpression of the MYC transcription factor being one of the most studied (9, 10). Therefore, there is an interest in characterizing metabolic changes associated with endocrine resistance, which may be developed into predictive markers to assist the patient at diagnosis and help the implementation of personalized follow-up schemes.

Targeted and untargeted metabolomics have been successfully applied to identify metabolite alterations in breast cancer, both in tissue and biofluids, to improve understanding of tumor metabolic pathways and unveil metabolic markers with diagnostic potential (11). Studies focused on changes in metabolism that are associated to acquired resistance to therapy have mainly considered cell line models of ER-positive/PR-positive, HER2-enriched and basal-like BC subtypes (12–16). Metabolic adaptations have been identified in cells that developed resistance to ET. In particular, comparison of the ET-resistant LCC9 cell line *vs.* the sensitive LCC1 cell line using integrated transcriptomics (microarray analysis) and metabolomics (GC-MS and UPLC-MS), identified increased levels of lysine, pyroglutamate, prostaglandin, 3,14-dihydro-15-keto prostaglandin F2-α (PGF2α), *N*-methyl-D-aspartate, L-2,3-dihydrodipicolinate, lysophosphatidylethanolamine (lysoPtE), lysophosphatidylcholine (lysoPtC), valine, α-(methylamino) isobutyrate, betaine, glutamate, hydroxybutyrate, hypotaurine, in tandem with decreased levels of specific unsaturated fatty acids (FAs) in resistant LCC9 cells, compared to sensitive LCC1 (12). NMR metabolomics of MCF-7 cells that acquired tamoxifen resistance registered increased levels of lactate, glycine, phosphocholine (PC) and glutamine, and decreased formate (13). In addition, long-term estrogen-deprived (LTED) cells (MM134 and SUM44 cell lines) were seen to activate FA/cholesterol metabolisms, with specific cholesterol metabolites (namely, 25-hydroxicholesterol and 27-hydroxicholesterol) driving cell proliferation (14). In the same study, significant upregulation of FA synthase (FASN) in LTED cells and variations in other key FA synthesis enzymes were detected. These results illustrate an important dependence on key enzymes in the FA/cholesterol pathways in endocrine-resistant ER-positive cells (14). Furthermore, selected amino acids (namely aspartate and glutamate) have been suggested to confer metabolic plasticity to both endocrine-resistant ER-positive cells and tumors (15). In addition, increased levels of α-ketoglutarate, citrate, glutamine, malate, oxaloacetate, succinate, uridine-5’-triphosphate (UTP) were found in LTDE cells compared with parental MCF7 cells (15). In a more recent report, metabolomics was combined with transcriptomics and metabolic flux experiments to address therapy resistance (16). The authors reported the metabolic adaptations of therapy resistant BC cells (MCF7-ESR1^Y537S,^ MCF7-ESR1^D538G^ and BT474) and sensitive cells (MCF7), disclosing that individual drugs impacted importantly on cell metabolism (16).

The role of PR in BC growth and response to therapy has gained much attention in the past years (5, 17). PR can modulate ERα transactivation of target genes, with genes like MYC or CCND1 containing regions regulated by ERα and PR, where both receptors physically interact to drive gene expression (18, 19). Thus, targeting PR stands as an alternative to treat specific subtypes of antiestrogen-resistant BC (5, 7). However, there is limited evidence (both *in vitro* and *in vivo*) about how progestins or antiprogestins impact on cell metabolism, and whether tumors that acquire resistance to ET, such as that involving mifepristone, alter their metabolism. Recently, the metabolomes of two HR-positive mouse mammary adenocarcinomas that rely on PR activation to sustain growth were compared, to show that different metabolic signatures characterize the metastatic and non-metastatic phenotypes (20). To our knowledge no metabolomic studies have analyzed metabolism changes in acquired antiprogestin resistance.

In the present study, the MPA-induced BC model was used for the characterization of polar metabolite changes, through untargeted Nuclear Magnetic Resonance (NMR) metabolomics, in neoplastic tissue *vs.* mammary tissue, and during the process through which tumors i) become independent of MPA for growth, and ii) subsequently become independent of HR signalling and acquire ET resistance. The results presented here are discussed under the light of putative biochemical mechanisms that characterize each tumor state/transition and potential metabolic biomarkers are advanced for each type of endocrine response.

## Materials and Methods

### Animal Model

**Figure 1:**
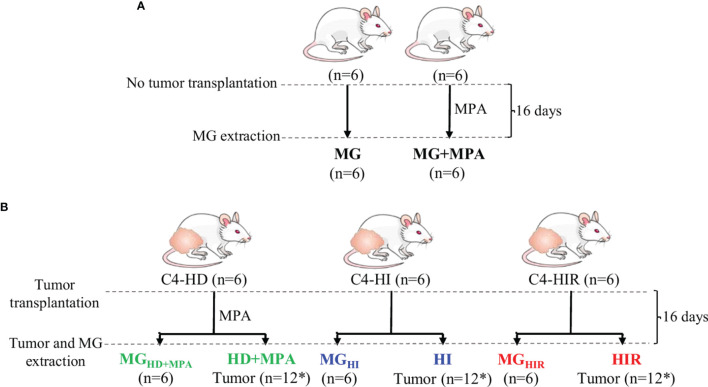
Experimental design and nomenclature of tissues from each group. **(A)** Two-month-old healthy, virgin female mice were divided into two groups, one implanted with 20 mg depot MPA and the other one left without depot (control). After 16 days, the mammary gland (MG) tissue was excised for analysis (MG+MPA and MG); and **(B)** the syngeneic tumors C4-HD (hormone-dependent; HD), C4-HI (hormone-independent; HI) and C4-HIR (hormone-independent and endocrine therapy-resistant; HIR) were implanted in the right and left inguinal flanks of 2-month-old virgin female mice. The mice bearing C4-HD tumors were also implanted with 20 mg MPA depot. The tumors (HD, HI and HIR) and MG tissue from the same animal (MG_HD+MPA,_ MG_HI_ and MG_HIR_) were excised and analysed. *Indicates that 12 tumors were obtained from six mice that were implanted on both inguinal flanks.

The mouse mammary ductal adenocarcinomas C4-HD, C4-HI and C4-HIR, all expressing ER and PR, from the MPA-induced model were used (21–23). Briefly, the parental tumor line, C4-HD, was induced by treatment of BALB/c female mouse with MPA depot and always transplanted with MPA to sustain growth (hormone-dependent; HD growth). Through successive passaging into mice without MPA the hormone-independent (HI) variant was established (C4-HI). Both C4-HD and C4-HI tumors regress if PR activation is blocked (either by MPA withdrawal in the case of C4-HD, or PR inhibition with antiprogestins (C4-HD and C4-HI). Through selective pressure, the C4-HIR (hormone-independent and therapy resistant) tumor line was obtained, which grows in the presence of the antiprogestins mifepristone and onapristone (21). In this work, each tumor line was implanted subcutaneously in the right and left inguinal flanks of 2-month-old BALB/C virgin female mice (n=6 mice and, thus, n=12 tumors) and allowed to grow until they reached a size of 30-40 mm^2^. During this time the animals were fed *ad libitum* and were kept under a 12 h light/dark cycle. Tumor samples and axial mammary gland (MG) tissue from the same animal were excised and immediately frozen in liquid nitrogen. In parallel, a group of mice (n=6) without tumors were grown in identical conditions (+/- 20 mg MPA depot), to obtain healthy MG tissue for comparison (**Figures 1A, B**). The mice were maintained at the Animal Facility at the Instituto de Biología y Medicina Experimental (IByME) of Buenos Aires, in Argentina. All animal procedures were performed at the IByME Animal facility and approved by the local Institutional Animal Care and Use Committee (Approval no. 030/2016, dated 24 June 2016), complying with regulatory standards of animal ethics.

### Sample Preparation and NMR Spectroscopy

Both tumor and MG tissue were ground using a pestle and mortar, while kept in liquid nitrogen. All tissue samples (average weight of 50 mg) were extracted using methanol: chloroform: water (1:1:0.75) (24) and the polar phase was separated for analysis. In brief, ground tissue samples were transferred to an eppendorf tube, followed by the addition of 500 µL of cold 80% methanol, 400µL of cold chloroform and 200 µL of cold Mili-Q water, and vortex homogenisation for 60 s. Samples were left to rest on ice for 10 minutes and then centrifuged (8,000 rpm, 5 min, 23°C). Polar phases were separated, vacuum-dried and stored at -80°C until analysis. At the time of NMR acquisition, the dried aqueous extracts were suspended in 600 µL of 100 mM sodium phosphate buffer (pH 7.4, in D_2_O containing 0.25% 3-(trimethylsilyl)-propionic-2,2,3,3-d4 acid (TSP) for chemical shift referencing), homogenized, and 550 µL were transferred to 5mm NMR tubes.

All NMR spectra were acquired on a Bruker AVANCE III spectrometer (Rheinstetten, Germany) operating at 500.13 MHz for proton. Standard 1D ^1^H NMR spectra of aqueous extracts were recorded at 298 K with water pressaturation, using the “noesypr1d” pulse program (Bruker library), with 2.34 s acquisition time, 2 s relaxation delay, 512 scans, 7002.801 Hz spectral width, and 32 k data points. Each free-induction decay was zero-filled to 64 k points and multiplied by a 0.3 Hz exponential function prior to Fourier transformation. After acquisition, spectra were manually phased, baseline-corrected, and chemical-shift referenced to TSP. For selected samples, two-dimensional NMR spectra, namely ^1^H-^1^H TOCSY (Total Correlation Spectroscopy) and ^1^H-^13^C HSQC (Heteronuclear Single Quantum Coherence) spectra were also recorded to support spectral assignment. Peak assignment was also based on comparison with data available on the Bruker BBIOREFCODE spectral database and the human metabolome database (HMDB) (25), as well as on existing literature.

### Statistical Analysis

Prior to multivariate analysis (MVA), the 1D ^1^H NMR spectra were converted to data matrices (AMIX-viewer 3.9.14, BrukerBiospin, Rheinstetten, Germany), excluding the suppressed water region (4.63-5.02 ppm) and residual methanol signals (3.35-3.36 ppm). The spectra were aligned by recursive segment-wise peak alignment (RSPA) (Matlab 8.3.0, The MathWorks Inc., Natick, Massachusetts, USA) and normalized to spectral total area, to account for different sample weights. 1D spectra data matrices were then scaled to unit variance (UV) and MVA was carried out using principal component analysis (PCA) and partial least-squares discriminant analysis (PLS-DA) (SIMCA-P 11.5; Umetrics, Umeå, Sweden). Results were visualized through factorial coordinates (‘scores plots’) and factorial contributions (‘loadings plots’), the latter colored according to variable importance to the projection (VIP). PLS-DA models were considered statistically robust for predictive power (Q^2^) values ≥ 0.5. For each relevant resonance, peak areas were integrated in the raw spectra (Amix 3.9.5, Bruker BioSpin, Rheinstetten, Germany), normalized to total spectral area and associated effect size (ES) values and statistical significance were calculated (p-value assessed by the student’s *t* test, R-statistical software, R Foundation for Statistical Computing, Vienna, Austria). Significant changes in metabolite levels (p < 0.05) were identified and p-values underwent the False Discovery Rate (FDR) correction for multiple comparisons based on the Benjamini and Hochberg method (26).

## Results

### Overall Metabolic Differences Between Tumors and MG Tissue

**Figure 2:**
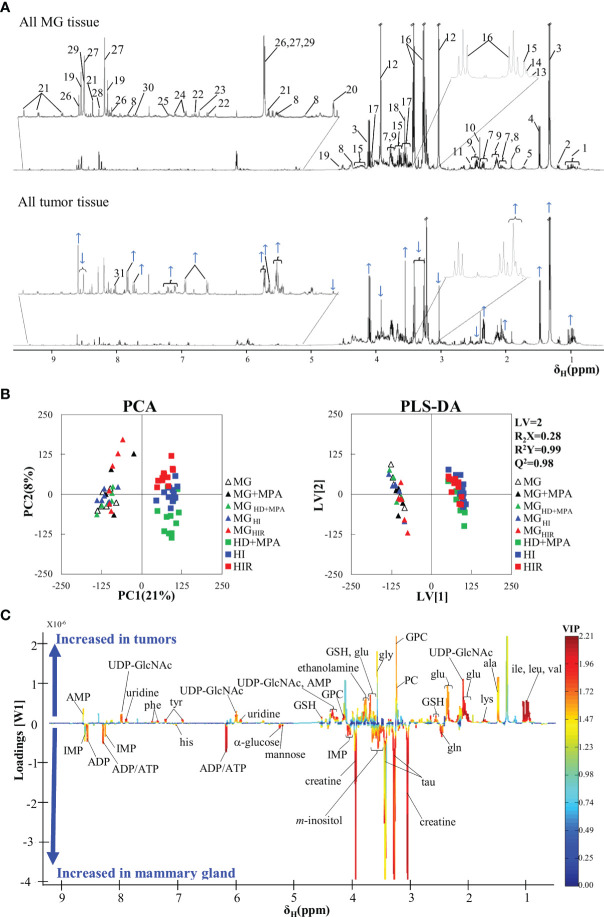
Average ^1^H NMR (500 MHz) spectra of aqueous extracts, PCA and PLS-DA scatter plots and corresponding loadings plots. **(A)** Average ^1^H NMR spectra of aqueous extracts of all tumors (top) and mammary gland (MG) samples (bottom). Peak assignments: 1: isoleucine/leucine/valine, 2: 3-HBA (3-hydroxybutyrate), 3: lactate, 4: alanine, 5: lysine, 6: acetate, 7: glutamate, 8: UDP-GlcNAc (UDP-*N*-acetylglucosamine), 9: glutamine, 10: succinate, 11: GSH (reduced glutathione), 12: creatine, 13: choline, 14: PC (phosphocholine), 15: GPC (glycerophosphocholine), 16: taurine, 17: *m*-inositol, 18: glycine, 19: IMP (inosine monophosphate), 20: α-glucose, 21: NAD^+^ (oxidized nicotinamide adenine dinucleotide), 22: tyrosine, 23: histidine, 24: phenylalanine, 25: uracil, 26: AMP (adenosine monophosphate), 27: ADP (adenosine diphosphate), 28: inosine, 29: ATP (adenosine triphosphate), 30: uridine 31: Un_8.12_: unassigned metabolite at δ 8.12; **(B)** PCA and PLS-DA scatter plots of all tumor samples (n = 36) *vs.* all MG samples (n = 30); and **(C)** corresponding PLS-DA loadings plots for the model in B) (right).

The average ^1^H NMR spectra of aqueous extracts from MG tissue and all murine MPA-induced mammary tumors are shown in **Figure 2A**. A total of 44 polar metabolites were identified overall, almost all detected in both MG and tumor tissue (**Table S1**). Compared to previous NMR reports of aqueous extracts from mouse and rat mammary tumors (20, 24, 27) and high-resolution magic angle spinning (HRMAS) NMR of human breast tumor tissue (28–30), all metabolites hereby identified in the MPA-induced tumors have been observed previously in different types of BC tissue, with the exception of adipate, ascorbate and hypoxanthine, which could not be detected here. Visual inspection of the average spectra of tumors and MG tissue (**Figure 2A**) suggests that tumors seem to exhibit qualitatively higher levels of lactate (peaks 3), some nucleotides (at *ca.* 6 ppm and higher), some amino acids [e.g. branched chain amino acids, branched-chain amino acids (BCAA) (peaks 1), alanine (peak 4), glutamate (peaks 7), glycine (peak 18), phenylalanine (peaks 24), and tyrosine (peaks 22)], glycerophosphocholine (GPC, peak 15), as well as decreased levels of creatine (peaks 12), glucose (peak 20), taurine (peaks 16) and glutamine (peaks 9). With the aim of statistically validating these (and possible other) variations, PCA was carried out, revealing effective group separation between all tumors and all MG tissue samples, and showing clear intra-group separation between HD, HI and HIR tumors (**Figure 2B**). The PLS-DA scores scatter plot of all tumors *vs* MG tissue (**Figure 2B**) was significantly robust (high predictive power, expressed by Q^2^ = 0.98), maximizing separation between all MG samples and all tumors (thus blurring the separation between tumor types, clearly seen in PCA). The corresponding loadings plot (**Figure 2C**) identified a large number of signals that were increased or decreased in tumors, compared with MG tissue (positive and negative signals, respectively). Indeed, a total of just over 30 metabolites and 4 still unassigned resonances were found to vary significantly between all mammary tumors (irrespective of their endocrine response) and MG tissue (independently of whether they were obtained from healthy or tumor-bearing animals) (**Table S2**). Clear changes comprise increased glutamate/glutamine ratio, increased alanine, BCAA, reduced glutathione (GSH) and aromatic amino acids, along with decreased taurine and creatine; decreased ATP and/or ADP and increased uridine diphosphate *N*-acetylglucosamine (UDP-GlcNAc) and uridine; increased total choline (GPC; in particular); increased lactate and reduced glucose and mannose; increased acetone and decreased inositol species. Notably, all variations were found to remain statistically significant upon FDR correction and exhibited low p-values, which demonstrate their robustness. However, these strong overall differences mask specific changes related to tumor endocrine response, as will be addressed in the next section.

In addition, it could be argued that MPA treatment or the origin of MG tissue (healthy or tumor-bearing mice) could influence metabolic profile and, hence, work as a confounder in this study. However, multivariate analysis results (**Figures 2B, C**) suggested that no significant differences occur between the metabolic profiles of i) MPA-treated and non-treated MG tissues, and ii) MG tissue from healthy animals and from tumor-bearing animals. Indeed, the absence of a significant MPA impact on tissue metabolic profile was evidenced by both PCA and PLS-DA (results not shown), the latter revealing that MPA-treated MG may show only weak increasing tendencies in GPC and GSH (effect sizes 1.36 ± 1.26, p-value 0.041; and 1.45 ± 1.27, p-value 0.015, respectively). In addition, MG from tumor-bearing mice (compared to that of healthy animals) only showed small variations in glutamine and mannose (effect sizes 1.09 ± 0.78, p-value 0.012; and -0.98 ± 0.77, p-value 0.025, respectively). The above weak tendencies were found to be negligible compared to the changes affecting the same metabolites for different tumor groups, as will be described below.

### Metabolite Differences Between Hormone-Dependent (HD), Hormone-Independent (HI) and Hormone-Resistant (HIR) Tumors

**Figure 3:**
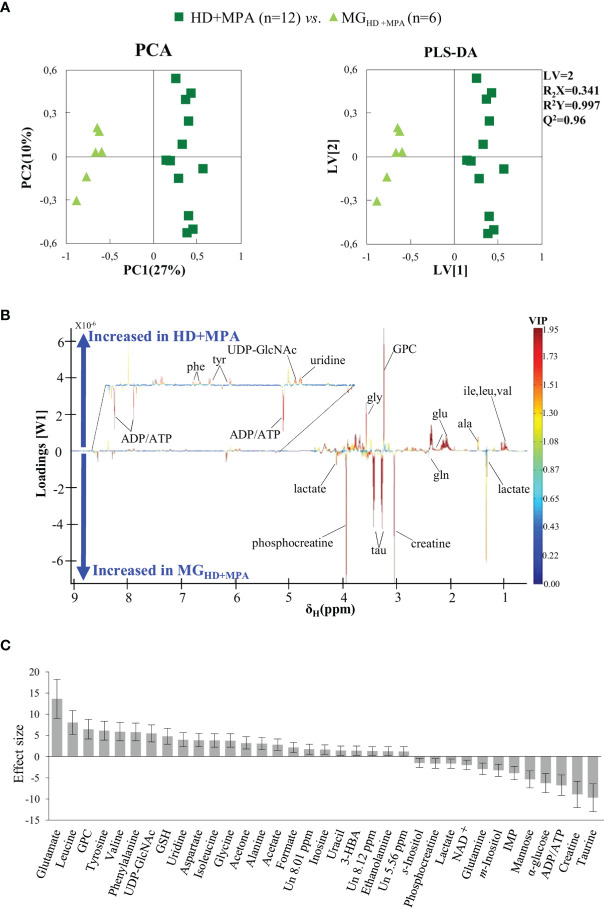
PCA and PLS-DA scatter plots, loadings plots and metabolic variations in the first step of tumor progression. **(A)** PCA and PLS-DA of HD+MPA tumors (n = 12) *vs.* MG_HD+MPA_ tissue (n = 6); **(B)** PLS-DA loadings plots; three-letter code used for amino acids and all other abbreviations defined as in **Figure 2** caption; and **(C)** metabolic variations reported in the transition from MG_HD+MPA_ tissue to HD+MPA tumors expressed by effect-size (ES), with corresponding error.

Unsupervised PCA analysis of the three tumor types showed a discernible separation of the three sample groups in PC1, further confirmed by PLS-DA (**Figure S1**), reflecting significantly distinct metabolic profiles of the respective polar extracts. Pairwise comparison of HD+MPA tumors and MG samples obtained from the same animals (MG_HD+MPA_) (**Figure 3**) produced robust group separations in both PCA and PLS-DA (Q^2^ = 0.96) scatter plots, along with a loadings plot rich in high VIP signals (**Figure 3B**), where positive and negative peaks reflect metabolites that are increased and decreased in tumors, respectively. Integration and subsequent univariate statistical analysis unveiled relevant changes in the levels of 14 amino acids and derivatives: GSH, creatine and phosphocreatine (PCr), 8 nucleotide-related metabolites, 2 precursors of membrane lipids (ethanolamine and GPC), 4 organic acids (acetate, formate, lactate and 3-hydroxybutyrate, 3-HBA), 2 sugars (glucose and mannose), 3 other compounds (ketone body acetone, and *m-* and *s-*inositols) (**Table 1**). Indeed, all metabolite variations identified (except for one unassigned resonance) remained significant after FDR correction (* in **Table 1**). **Figure 3C** illustrates that the largest changes, as viewed by effect size, comprise: i) increases in glutamate, all three BCAA, UDP-GlcNAc, GPC and GSH; and ii) decreases in taurine, creatine, ADP and/or ATP, glucose, among others (notably, only a small decrease is noted for lactate in HD tumors).

**Table 1:** Metabolite changes in aqueous extracts of pairwise group comparisons.

Compound	HD+MPA *vs*. MG_HD+MPA_		HI *vs*. HD+MPA		HIR *vs*. HI	
	ES ± error	*p-*value	ES ± error	*p-*value	ES ± error	*p-*value
**Amino acids and derivatives**						
Alanine	3.08± 1.41	1.08E-04*	1.78 ± 0.94	2.74E-04*	-1.72 ± 0.94	6.56E-04*
Aspartate	3.86 ± 1.60	1.08E-04*	-1.09 ± 0.86	2.91E-03*	-1.28 ± 0.88	1.03E-04*
Creatine	-8.92 ± 4.56	1.08E-04*	-2.07 ± 0.99	3.33E-05*	—	—
Glutamate	13.64 ± 3.95	1.08E-04*	-6.84 ± 2.09	7.40E-07*	-3.68 ± 1.31	7.40E-07*
Glutamine	-2.90 ± 1.36	2.16E-04*	-3.17 ± 1.20	7.40E-07*	-3.56 ± 1.29	7.40E-07*
GSH	4.79 ± 1.85	1.08E-04*	1.53 ± 0.91	1.43E-03*	-1.81 ± 0.95	4.96E-04*
Glycine	3.75 ± 1.57	1.08E-04*	-2.02 ± 0.98	2.74E-04*	-3.10 ± 1.19	7.40E-07*
Isoleucine	3.80 ± 1.58	1.08E-04*	—	—	—	—
Leucine	8.07 ± 2.81	1.08E-04*	1.25 ± 0.87	1.45E-02*	-1.39 ± 0.89	3.64E-03*
Phenylalanine	5.79 ± 2.13	1.08E-04*	1.31 ± 0.88	5.56E-03*	—	—
Phosphocreatine	-1.60 ± 1.11	2.45E-02*	-1.44 ± 0.90	2.91E-03*	-4.23 ± 1.44	7.40E-07*
Taurine	-9.65 ± 3.30	1.08E-04*	-3.18 ± 1.20	8.88E-06*	1.81 ± 0.95	6.56E-04*
Tyrosine	6.13 ± 2.23	1.08E-04*	—	—	-0.85 ± 0.84	4.49E-02
Valine	5.88 ± 2.16	1.08E-04*	—	—	—	–
**Nucleotides and derivatives**						
AMP	—	—	—	—	1.10 ± 0.86	2.84E-02
ADP/ATP	-6.76 ± 2.41	1.08E-04*	1.74 ± 0.94	2.74E-04*	-1.11 ± 0.86	5.56E-03*
IMP	-3.86 ± 1.60	8.31E-04*	1.01 ± 0.85	1.21E-02*	2.18 ± 1.01	2.22E-05*
Inosine	1.64 ± 1.12	8.31E-04*	-1.65 ± 0.93	1.44E-04*	-1.13 ± 0.86	1.21E-02*
NAD^+^	-1.98 ± 1.17	4.74E-03*	—	—	—	—
UDP-GlcNAc	5.46 ± 2.04	1.08E-04*	5.89 ± 1.85	7.40E-07*	-2.70 ± 1.11	2.22E-05*
Uracil	1.45 ± 1.09	2.62E-02*	—	—	2.50 ± 1.07	7.17E-05*
Uridine	3.98 ± 1.63	1.08E-04*	-1.21 ± 0.87	1.21E-02*	—	—
**Choline compounds and other membrane lipids precursors**						
Choline	—	—	-0.91 ± 0.84	2.05E-02*	1.00 ± 0.85	3.32E-02
Ethanolamine	1.29 ± 1.07	3.23E-03*	-1.71 ± 0.94	2.22E-05*	—	—
GPC	6.41 ± 2.31	1.08E-04*	-2.35 ± 1.04	5.18E-06*	-4.70 ± 1.55	7.40E-07*
PC	—	—	1.02 ± 0.85	1.45E-02*	-1.74 ± 0.94	7.17E-05*
**Organic acids**						
Acetate	2.80 ± 1.34	2.16E-04*	-1.53 ± 0.91	1.83E-03*	1.06 ± 0.85	2.42E-02*
Formate	2.15 ± 1.21	1.08E-04*	-1.46 ± 0.90	3.64E-03*	—	—
Lactate	-1.62 ± 1.11	4.74E-03*	5.55 ± 1.76	7.40E-07*	4.09 ± 1.41	7.40E-07*
Succinate	—	—	-1.71 ± 0.94	3.71E-04*	—	—
3-HBA	1.40 ± 1.08	1.35E-02*	—	—	1.48 ± 0.90	1.83E-03*
**Sugars**						
Mannose	-5.38 ± 2.01	8.63E-04*	—	—	—	—
α-glucose	-6.24 ± 2.26	1.08E-04*	—	—	—	—
**Other compounds**						
Acetone	3.20 ± 1.43	1.08E-04*	-1.40 ± 0.89	4.51E-03*	1.13 ± 0.86	2.05E-02*
*m*-Inositol	-3.23 ± 1.44	1.08E-04*	—	—	—	—
*s-*Inositol	-1.51 ± 1.10	3.23E-03*	—	—	—	—
**Unassigned**						
Un _5.56 ppm_	1.21 ± 1.06	3.80E-02*	3.61 ± 1.30	8.88E-06*	-2.02 ± 0.98	1.44E-04*
Un _7.69 ppm_	—	—	0.84 ± 0.83	3.87E-02	—	—
Un _8.01 ppm_	1.75 ± 1.13	2.16E-04*	-1.66 ± 0.93	3.71E-04*	—	—
Un _8.12 ppm_	1.31 ± 1.07	2.45E-02*	1.36 ± 0.89	4.51E-03*	2.62 ± 1.09	1.48E-06*

3-HBA, 3-hydroxybutyrate; AMP, adenosine monophosphate; AXP, overlap of ADP/ATP; IMP, inosine monophosphate; GPC, glycerophosphocholine; GSH, reduced glutathione; NAD^+^, nicotinamide adenine dinucleotide; PC, phosphocholine; UDP-GlcNAc, uridine diphosphate N-acetylglucosamine; Un, unsigned resonance; *Variations remaining significant after FDR correction.

**Figure 4:**
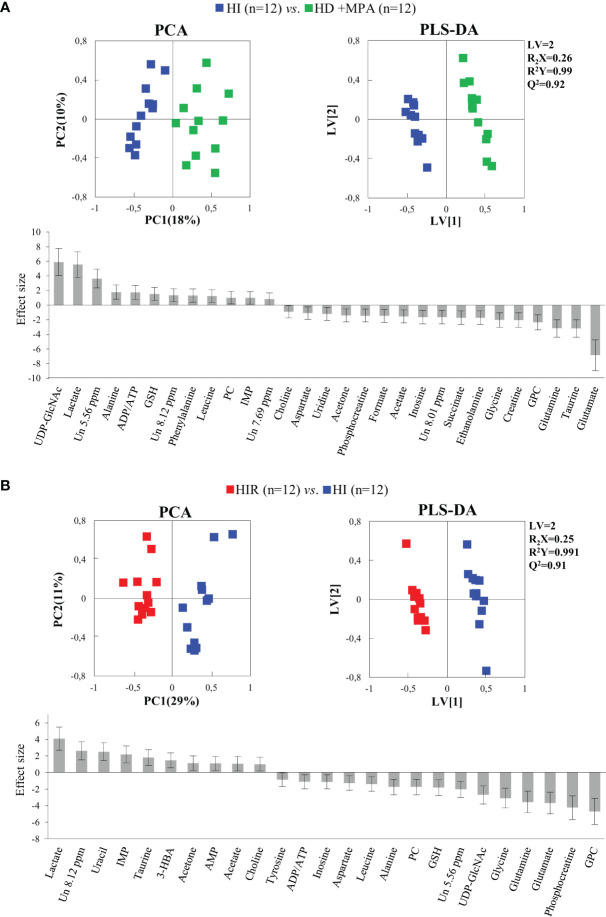
PCA and PLS-DA scatter plots and metabolic variations for acquisition of hormonal independence and acquisition of treatment resistance. **(A)** PCA and PLS-DA of HI tumors (n = 12) *vs.* HD+MPA tumors (n = 12) and corresponding metabolic variations expressed by ES; **(B)** PCA and PLS-DA of HIR tumors (n = 12) *vs.* HI tumors (n = 12) and corresponding metabolic variations expressed by ES. All abbreviations defined as in **Figure 2** caption.

Regarding HI tumors, compared to HD+MPA tumors (**Figure 4A**, top), once again multivariate analysis indicated robust group separation (Q^2 ^= 0.92 for PLS-DA). Regarding amino acids, BCAA are hardly changed (note the absence of changes in isoleucine and valine, with a weak increase in leucine, p-value 0.0145), whereas inversions of variation direction were noted for aspartate (↓), glutamate (↓) (the latter now exhibiting the largest decrease, **Figure 4A**, bottom) and glycine (↓). In addition, other variation inversions affected ADP and/or ATP (↑), inosine and uridine (↓), ethanolamine (↓), GPC (↓), acetate (↓), formate (↓), and acetone (↓). Notably, UDP-GlcNAc exhibited the largest increase, followed by lactate (**Figure 4A**, bottom) and, while new weaker changes were noted for choline, PC and succinate, other metabolite levels remained unaltered between HI and HD+MPA tumors, namely, NAD^+^, uracil, 3-HBA, sugars (mannose and glucose) and inositols.

Metabolic profile differences between HI and HIR tumors also became clear in both PCA and PLS-DA scores scatter plots (**Figure 4B**, top), although now defining a partially distinct signature for the latter (**Table 1**), where BCAA remained unchanged (except for a slight decrease in leucine), as were creatine, phenylalanine and inositol species. Reversed changes in HIR tumors, compared to HI tumors, affected alanine (↓), GSH (↑), taurine (↑), ADP/ATP (↓), UDP-GlcNAc (↓), PC (↓), acetate (↑), and acetone (↑). Importantly, lactate increase is seen to correspond to the largest positive variation (thus expressing an important continuing increase from HD to HI and to HIR tumors), while significant decreases affected GPC, PCr, glutamate and glutamine (**Figure 4B**, bottom). The effect size values listed in **Table 1** are also illustrated in a heatmap (**Figure S2**), where it becomes visually clear that HD tumors exhibit changes, compared to MG tissue of the same animals, in a relatively larger number of metabolites in most families (32 identified metabolites), with statistical relevance described by p-values between 0.05 and 0.001. HD to HI, and HI to HIR transitions are accompanied by changes in 25 and 23 metabolites, respectively, in some cases with relatively stronger statistical relevance (p-value < 0.0001).

**Figure 5:**
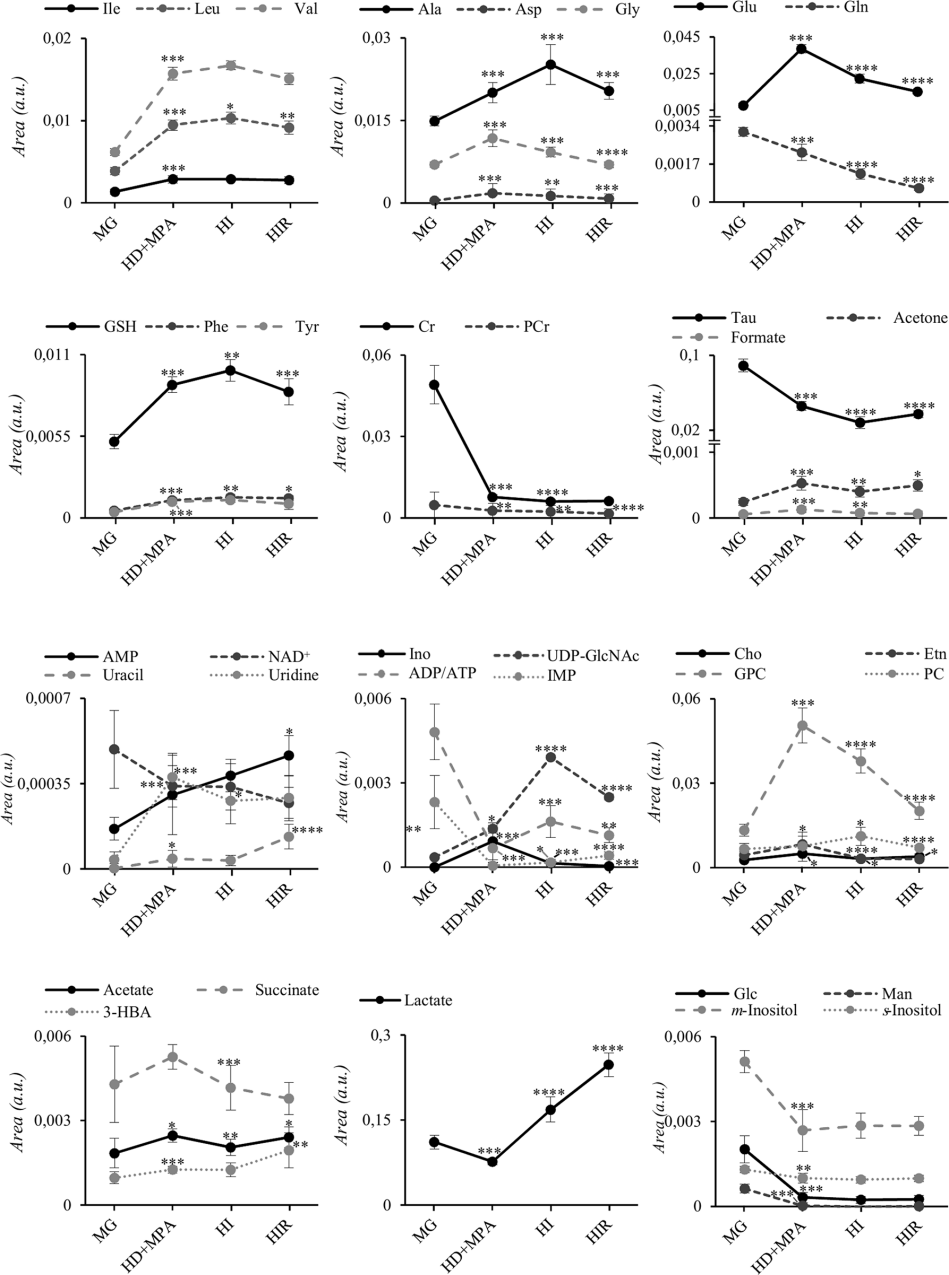
Metabolite trajectories throughout tumor stage progression. Peak areas corresponding to each metabolite were normalized by total spectral area. MG, mammary gland from healthy mice; HD+MPA, hormone-dependent tumor; HI, hormone-independent tumor; HIR, hormone-independent and therapy resistant tumor. *p-value < 0.05; **p-value < 0.01; ***p-value < 0.001; ****p-value < 0.0001. Three-letter code used for amino acids; Cr, creatine; Etn, ethanolamine; Glc, glucose; Ino, inosine; Man, mannose; PCr, phosphocreatine. All other abbreviations are defined as in **Figure 2** caption.

**Figure 6:**
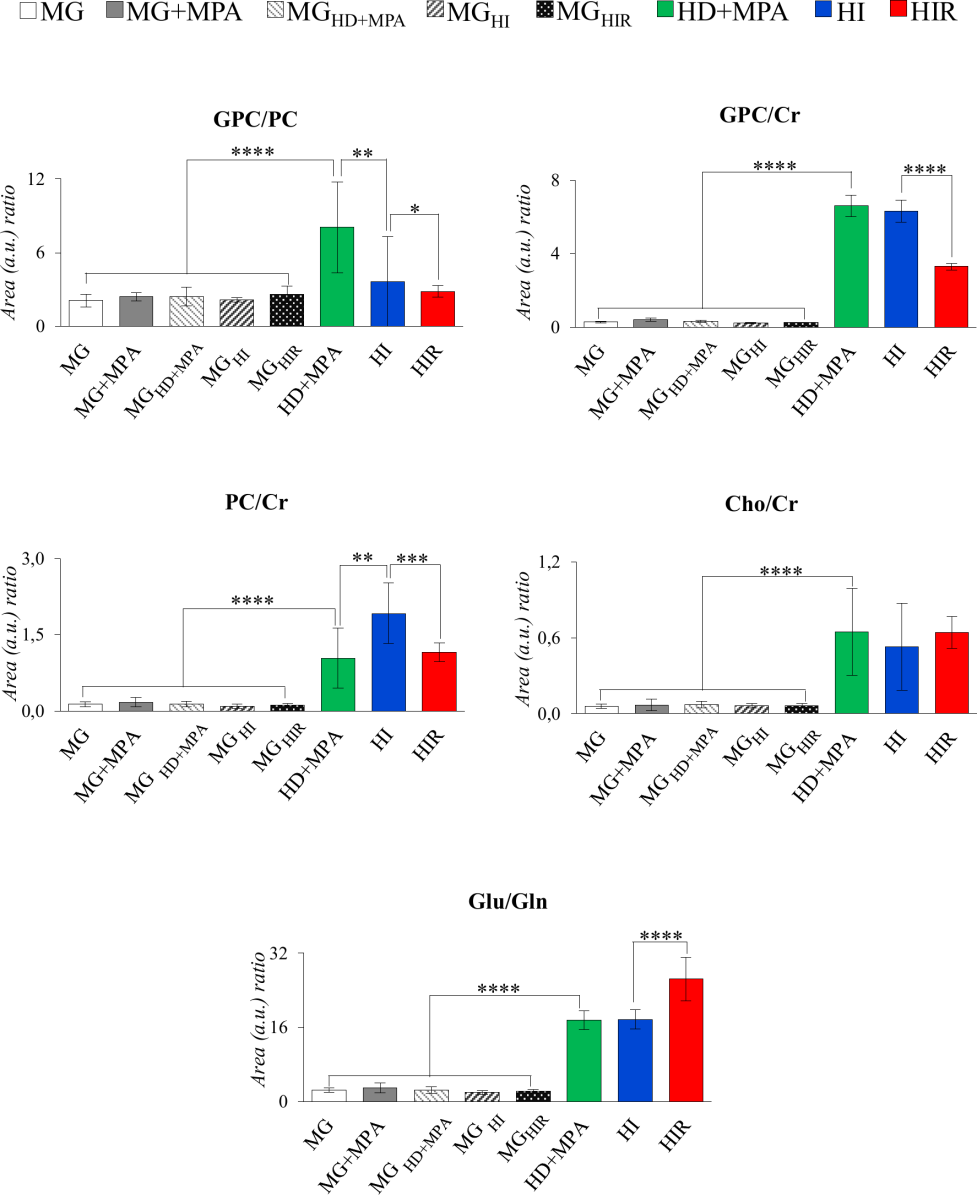
Graphical representation of selected metabolite ratios. All ratios were obtained from the average normalized peak areas. All abbreviations defined as in **Figure 2** caption. Asterisks represent the statistical relevance of a certain group compared to the previously represented one: *p-value < 0.05; **p-value < 0.01; ***p-value < 0.001; ****p-value < 0.0001.

The trajectories followed by each relevant metabolite are shown in **Figure 5** to illustrate firstly, that all tumors may be distinguished from MG tissue mainly by significant increases in BCAA, alanine, glutamate, GPC and GSH, and decreases in creatine, taurine, ATP and/or ADP, IMP, glucose, mannose and *m-*inositol (while other significant changes characterize lower abundance metabolites, such as glutamine, phenylalanine, tyrosine, uridine and UDP-GlcNAc, **Table S2**), together with relatively higher GPC/Cr, PC/Cr, Cho/Cr and Glu/Gln ratios (**Figure 6**) [illustrative ratios discussed in previous tissue studies (31–35)]. Therefore, these changes constitute a differentiating metabolite signature of these mammary carcinomas relatively to MG, independently of their endocrine response.

In addition, tumor groups with different endocrine responses may be clearly distinguished between each other in terms of single metabolite levels (**Figure 5**), as follows: i) HD tumors, with higher levels of glycine, glutamate, inosine and GPC, and lower levels of lactate; ii) HI tumors, with higher levels of alanine, UDP-GlcNAc, ATP and/or ADP, and lactate levels intermediate between those of HD and HIR tumors, iii) HIR tumors, with lower levels of GPC and glutamate (though remaining higher than MG), intermediate levels of UDP-GlcNAc (between HD and HI) and maximum levels of lactate. In particular, the relative levels of glutamate, UDP-GlcNAc, GPC, lactate and ATP and/or ADP seem to be capable of differentiating all three types of tumors considered in this study. Further to single metabolite levels, specific metabolite ratios also add to the specific signatures of each tumor type (**Figure 6**), with i) HD tumors having higher GPC/PC ratios than all other tumors and MG samples, ii) HI tumors having higher PC/Cr ratios than all other samples, and iii) HIR tumors having GPC/Cr ratios intermediate between those in other tumors and MG samples, as well as particularly high levels of Glu/Gln (mainly due to an order of magnitude decrease in glutamine levels, and in spite of the noted decrease in glutamate, **Figure 5**).

## Discussion

Cancer cells can reprogram their metabolism to support proliferation, invasion, and resistance to anti-cancer therapy (36, 37). This allows them to survive in stressful conditions and reflects the acquisition of oncogenic mutations and epigenetic modifications. Understanding the metabolite changes during cancer progression is relevant for diagnosis, follow-up and treatment, hence, this study addresses three mammary invasive ductal adenocarcinoma tumour lines that express similar levels of ER and PR but respond differently to ET (21), with the aim of characterising the metabolic profiles of neoplastic tissue compared to normal tissue (MG), and disclosing metabolic signatures discriminative of HD, HI and HIR tumors. As expected, metabolite profile differences were of larger magnitude between HD tumours and MG, than during the progression towards hormone-independence and therapy resistance. Nevertheless, interesting characteristic metabolite changes could be identified for each tumour line.

Compared to MG, all tumors, irrespective of their endocrine response, shared higher levels of BCAA, alanine, glutamate, GSH and GPC, and lower levels of creatine, taurine, ATP and/or ADP, IMP, glucose, mannose and *m-*inositol. Changes in lower concentration metabolites e.g., glutamine, phenylalanine, tyrosine, uridine and UDP-GlcNAc were also observed. It is important to note that the average metabolite changes described above may mask finer characteristics of each different tumor type; however, this group of metabolites may be useful in differentiating healthy and tumoral tissues. Aerobic glycolysis is a hallmark of many cancers (36) and, in this model, there is a high glucose and mannose demand by proliferating cancer cells to sustain glycolysis. Therefore, glutamate dehydrogenase activity would then be responsible for the production of TCA cycle intermediate α-ketoglutarate, consistently with the observed high alanine levels and slightly high lactate levels. Note that lactate levels are strongly tumor type dependent (see below) and only a weak overall increase is noted when all tumors are grouped together. BCAAs are essential amino acids and, thus, need to be internalized from the tumor microenvironment or obtained through protein degradation. Tumor cells express high levels of specific amino acid transporters according to the specific tumor type (38). SLC7A5 (BCAA, Phe and Tyr transporter) is a prognostic factor for breast cancer (39) and is included in the Mammostrat prognosis prediction test for ER-positive breast cancer tumors (40); while, SLC6A14 (transporter of neutral and cationic amino acids) is highly expressed in ER-positive breast cancer tissue and cell lines, and its transcription is under Erα and MYC regulation (41). BCAA activate the mTORC1 pathway to stimulate protein synthesis, tumor growth and survival, but can also be metabolized into branched-chain α-keto acids in a process involving conversion of α-ketoglutarate to glutamate (catalysed by BCAT1 or BCAT2 BCAA transaminase activity), and/or further catabolized to acetyl-CoA and succinyl-CoA that feed into the TCA (42). Thus, we suggest that BCAA internalization in this model is possibly enhanced to feed into the TCA cycle, contributing to the higher levels of glutamate (through BCAA transaminase activity of BCAT enzymes), in tandem with glutaminolysis (through glutaminase, GLS). Indeed, BCAT1 levels have been shown to be elevated in various breast cancer tissues (including invasive carcinoma and intraductal carcinoma) as compared with normal breast tissue (43, 44). In addition, elevated GLS expression has been associated with high grade and metastatic breast cancer (38) and inhibition of GLS activity or gene expression prevents oncogenic transformation (45) and tumor growth (46). Interestingly, the MPA-induced tumors used in this study rely on ER/PR induction of the oncogene MYC to grow, with MYC inhibition resulting in cancer cell death and tumor regression (47, 48). MYC activates transcription of the SLC1A5a glutamine transporter to increase glutamine influx, also activating SLC6A14 (49) and GLS overexpression (49, 50). Glutamate may also help to sustain the high GSH levels in the tumors, to support anti-oxidative stress mechanisms through reactive oxygen species (ROS) detoxification, reducing oxidative damage to protect cells from apoptosis (51). These protective mechanisms may involve taurine (52, 53) and inositol species (54), thus justifying the decrease of these metabolites. On the other hand, the higher taurine and creatine levels in MG, compared to the tumors, could also possibly reflect the higher thermogenic capacity of adipocyte-rich MG tissue, since this tissue has been shown to contain brown adipose tissue (55, 56). Furthermore, the higher levels of creatine relatively to PCr in tumors may reflect the need of the former for ATP synthesis (57), whereas increased choline and choline-containing metabolites are characteristic of cancer tissue. Elevated levels of phosphocholine and glycerophosphocholine have been reported in most types of cancer confirming the importance of choline metabolism in cancer development (35). Choline compounds are usually associated with cell proliferation (58), choline metabolism being controlled by Erα (59) and MYC (60). In this tumor model, GPC increase is the main reason for the noted increased choline ratios (Cho/Cr, GPC/Cr, PC/Cr) and main contribution for higher total choline content. In relation to UDP-GlcNAc levels, we suggest that cancer cells upregulate the hexosamine biosynthetic pathway (HBP), with increased glucose and glutamine uptake as well as oncogenic signals mediated by Ras oncogenes (61) and mTORC2 (62) leading to higher UDP-GlcNAc levels. *N*-linked and *O*-linked glycosylation use UDP-GlcNAc as substrate to modify the activity of metabolic enzymes, transcription factors, and several signalling molecules (63). Furthermore, *N*-glycosylation has been proven to be elevated in elevated in breast cancer primary tumors, as well as in lymph node metastases and distant metastases compared to healthy mammary tissue (64).

**Figure 7:**
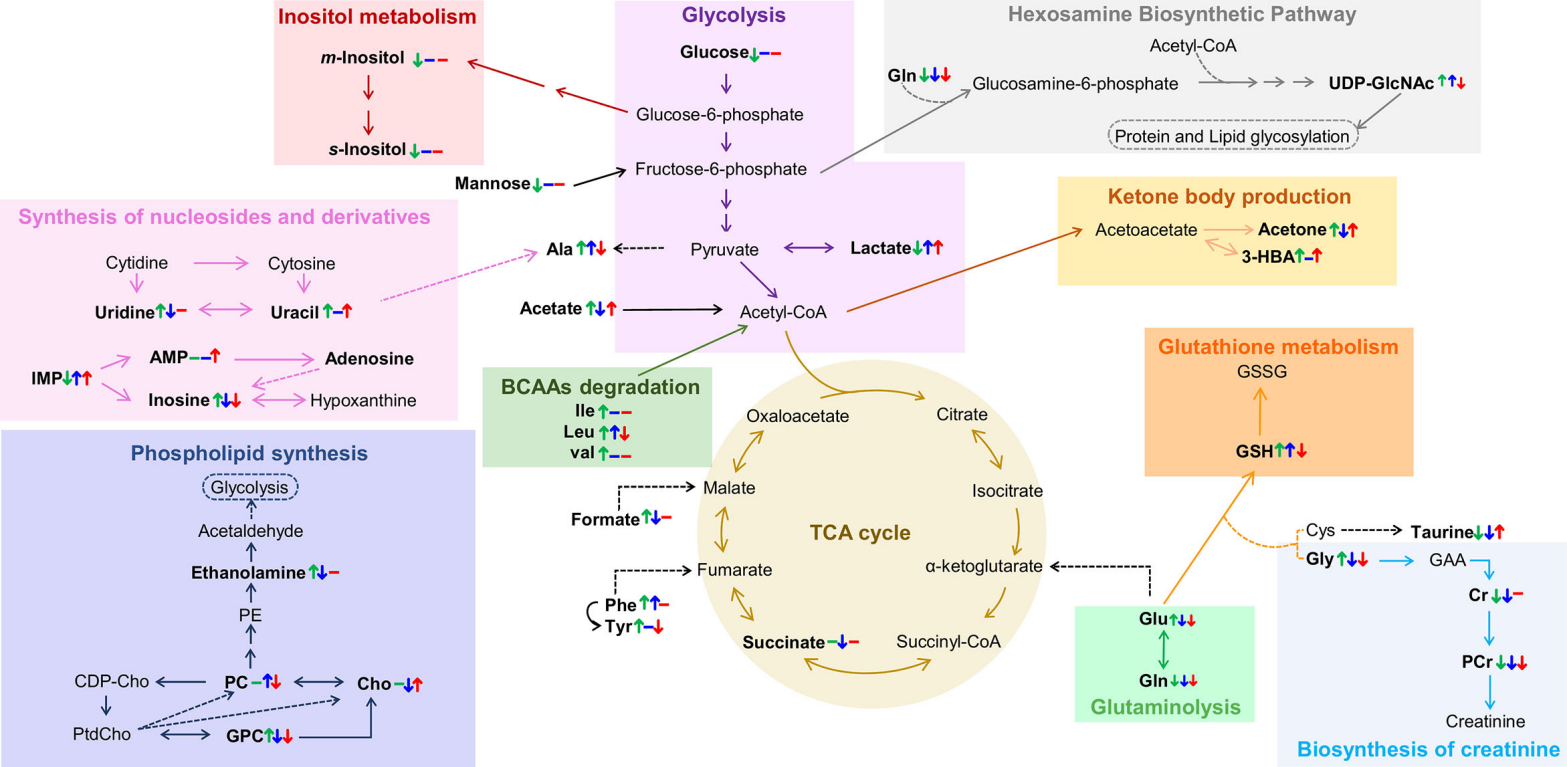
Metabolic pathways putatively identified as the main metabolic adaptations found between polar extracts of MG_HD+MPA_, HD+MPA, HI and HIR tissues. Metabolite names in bold identify compounds detected by NMR (irrespective of their variation). Colored arrows (↓↑) and dashes (-) indicated after a metabolite name illustrate variations corresponding to each pairwise comparison, following the order: HD+MPA *vs* MG_HD+MPA_ (left symbol), HI *vs* HD+MPA (middle symbol), and HIR *vs* HI (right symbol). Three letter code were used for amino acids; CDP-Cho, cytidine diphosphate-choline; GAA, guanidoacetate; PE, phosphoethanolamine; PtdCho, phosphatidylcholine, all other abbreviations defined as in **Figure 2** caption.

However, the main contribution of this work regards the metabolic distinction between tumors characterized by different endocrine responses, here designated by hormone-dependent, hormone-independent/sensitive to therapy and hormone-independent/resistant to therapy (HD, HI and HIR, respectively). In an attempt to illustrate the interplay of relevant active metabolic pathways determining the metabolic profile of each tumor type, **Figure 7** illustrates the main changes characterizing each comparison discussed above i.e., MG to HD tumors, HD to HI tumors, and HI to HIR tumors. The reasons for these changes in terms of enzyme activity adaptations remain to be established and this will be the scope of follow-up work. Interestingly, HD tumors show a non-glycolytic metabolic pattern, as glucose (and other sugars) are broken down more extensively than in MG and in the other tumor types but do not translate into higher lactate levels or ADP and/or ATP levels. On the contrary, the decrease of lactate and ADP and/or ATP suggests the absence of the Warburg effect and no particular enhancement of TCA cycle activity in HD tumors. It is possible, therefore, that glucose is preferentially used as precursor of protein/lipid glycosylation processes, through the synthesis of UDP-GlcNAc as explained above. In relation to glutamine metabolism, it appears that HD tumors maintain glutamine levels. The glutamine transporters SLC1A5 and SLC6A14 are highly expressed in ER-positive breast cancer as they are under transcriptional control of ER and MYC. Therefore, glutamine levels are maintained through thehosphn of these transporters to activate glutamate synthesis (glutamate reaches a maximum level in HD *vs* other tumors), as well as to feed the TCA cycle and produce GSH. Relatively high GPC levels (and high GPC/PC ratio) clearly distinguish HD tumors from the remaining types, GPC levels remaining above those characteristic of MG tissue. Indeed, the interplay of GPC and other membrane lipid precursors is clearly dependent on tumor type. As comparable degrees of cell proliferation are expected for all tumors as they were all excised in the exponential growth phase, and therefore hosphatidylcholine biosynthesis would be maintained relatively unchanged, these differences between tumors may relate to increasing capacity of organelles such as the endoplasmic reticulum (see below).

The development of HI tumors seems to involve the production of higher levels of lactate and alanine levels. These tumors are, thus, more glycolytic (increase in lactate) than HD tumors and MG. Aerobic glycolysis has been shown to be associated with the resistance to chemotherapy and ET resistance. In particular, tamoxifen resistance is associated with enhanced glycolysis in ER-positive breast cancer cells through activation of EGFR/MAPK pathway (65, 66), a pathway also responsible for ligand-independent Erα activation (37) In the MPA-induced tumor model, the FGF-2/FGFR-2 axis drives HI growth (67). This is mediated by ligand-independent Erα and PR activation, followed by their induction of MYC expression and was also verified in human breast cancer cells (48). FGFR signalling leading to MYC activation has been shown to induce glycolytic enzymes necessary for vascular development (68), while MYC itself also induces the expression of many glycolytic enzymes. Thus, increased glycolysis resulting from MYC-dependency is somewhat expected in this model. Additionally, the low glucose levels may be indicative, not only of enhanced glycolysis activity, but also of enhanced conversion (together with acetyl-CoA) into UDP-GlcNAc, which shows its highest level in HI tumors; consistently, uridine and uracil levels (needed for UTP biosynthesis later used for UDP-GlcNAc) are kept low in HI tumors, possibly to support the more significant glycosylation processes apparently characterizing such tumors. In agreement with our findings, a previous microarray comparing C4-HD and C4-HI tumours found upregulation of glutamine-fructose-6-phosphate aminotransferase 2 (GFPT2, which controls the flux of glucose into the hexosamine pathway) (22). The fact that UDP-GlcNAc is upregulated in HI tumors is very interesting as this is the precursor of O-glycosylation that has been shown to occur in ER and PR to promote hormone-independent activation (69, 70). HI tumors exhibit intermediate glutamate levels, which may indicate an enhanced uptake of this amino acid into the TCA cycle but also higher glutathione synthase (GSS) activity as these tumors also have higher GSH than the other tumor types. A statistically relevant increase in PC (and specifically high PC/Cr ratios) again reflects the distinct membrane metabolism characteristics of each tumor type. It is possible that at least some of the above distinguishing features between HD and HI tumors, e.g. altered amino acid metabolism (20, 71, 72), may be relatable to the higher metastatic behavior of the latter tumors (21), although such possibility requires further investigation.

Finally, the hormone-independent tumors which have become resistant to therapy, HIR, are the most glycolytic tumors, reaching the highest lactate levels, while maintaining glucose levels low. This strong glycolytic nature is consistent with the specifically high Glu/Gln ratio, due to a marked decrease in glutamine, identifying active glutaminolysis as an additional feature of HIR tumors. Therefore, and as mentioned previously, the metabolic dependency on glucose and glutamine that was clear for HI tumors and associated with resistance to ET and FGFR driven MYC expression, becomes enhanced in HIR tumors. Moreover, HIR tumors exhibit similar levels of GSH, taurine and *m*-inositol to other tumors, indicating similar extension of active anti-oxidative mechanisms also associated with ET resistance (73), however they are distinguished by the lowest glutamate levels (and low alanine) within the tumors, which possibly are consumed to produce GSH. UDP-GlcNAc is also upregulated in HIR but to a lesser extent than in HI tumors maintaining a relevant protein glycosylation activity; still, it is worth mentioning that endocrine resistant breast cancer cells increase their endoplasmic reticulum capacity (through the activation of unfolded protein response), which is one of the major cellular organelles where protein glycosylation occurs (74, 75). In HIR tumors, the lowest GPC levels (within the tumors) again seems to reflect a distinct status of membrane metabolism where lower GPC may be the result of higher phospholipid (phosphatidylcholine, main membrane constituent) synthesis. Again, potentially different metastatic status may relate to the signature differences described above, although such feature could not be evaluated for HIR tumors, due to their rapid growth and subsequent early sacrifice of the animals (before metastasis could occur or be detected) (76). This possible relationship between metabolite signatures and tumor metastatic status is of large interest in BC research and requires further investigation.

## Conclusions

Our results show, for the first time to our knowledge, that different endocrine response of MPA-induced breast tumors clearly relate to distinct metabolic signatures. Several pathways are observed to adapt to each of HD, HI and HIR status, mainly with the involvement of selected amino acids, nucleotides, choline compounds and sugars. In particular, the relative levels of glutamate, UDP-GlcNAc, GPC and lactate seem to be capable of differentiating all three types of tumors, with HIR tumors showing a relatively stronger glycolytic profile, specific membrane metabolism pattern and an extent of O-glycosylation processes which is intermediate to HD and HI tumors. While our results unveil the importance of metabolic adaptations and potential metabolic markers to distinguish different endocrine responses and, in particular, therapy resistance in breast tumors, the present study has some limitations, which should be addressed in future follow-up work, namely the use of relative metabolite levels instead of absolute concentrations (which are difficult to assess efficiently by NMR of complex mixtures but feasible by other more targeted analytical methods), and the awareness that the metabolic profiles identified here reflect the tumors as a whole, thus globally representing the heterogeneous population of cell types in the tumor microenvironment. The relationship between metabolic profile and cell distribution in both environments would be an interesting follow-up step in understanding the hereby presented metabolic adaptations and relationship to resistance. Furthermore, given the recognized relevance of the MPA-model to the human disease, the translation of these results to human subjects is a necessary step and a subject of ongoing work in our group.

## Data Availability Statement

The original contributions presented in the study are included in the article/**Supplementary Material**. Further inquiries can be directed to the corresponding authors.

## Ethics Statement

All animal procedures were performed at the IByME Animal facility and approved by the local Institutional Animal Care and Use Committee (Approval no. 030/2016, dated 24 June 2016), complying with regulatory standards of animal ethics.

## Author Contributions

RA: experimental design, data acquisition and processing; data analysis, writing of first draft, editing, and finalization of paper. VF: experimental design, sampling, and editing and finalization of paper. CaL: experimental design, sampling, and editing and finalization of paper. ClL: conceptualization, experimental design, sampling, editing and finalization of paper, and funding acquisition. LH: conceptualization, experimental design, data analysis, writing of first draft, and editing and finalization of paper, funding acquisition. AG: conceptualization, experimental design, data analysis, writing of first draft, editing and finalization of paper, and funding acquisition. All authors contributed to the article and approved the submitted version.

## Funding

This work was developed within the scope of the project CICECO-Aveiro Institute of Materials, UIDB/50011/2020, UIDP/50011/2020 & LA/P/0006/2020, financed by national funds through the FCT/MEC (PIDDAC). AG acknowledges the Portuguese National NMR Network (RNRMN), supported by FCT funds, and RA thanks RNRMN for her grant through the Doctoral Program in NMR applied to Chemistry, Materials and Biosciences – PTNMRPhD (PD/00065/2013). The authors also acknowledge financial support from the Portuguese Foundation for Science and Technology through projects UIDB/04501/2020, UIDP/04501/2020, MEDISIS (CENTRO-01-0246-FEDER-000018) and pAGE (CENTRO-01-0145-FEDER-000003) project cofounded through the Comissão de Coordenação e Desenvolvimento Regional do Centro and COMPETE 2020 program and European Union fund FEDER (LH). The authors also acknowledge the Agencia Nacional de Promoción Científica y Tecnológica, Argentina (ANPCYT); grant PICT 2017-2073 (CL).

## Conflict of Interest

The authors declare that the research was conducted in the absence of any commercial or financial relationships that could be construed as a potential conflict of interest.

## Publisher’s Note

All claims expressed in this article are solely those of the authors and do not necessarily represent those of their affiliated organizations, or those of the publisher, the editors and the reviewers. Any product that may be evaluated in this article, or claim that may be made by its manufacturer, is not guaranteed or endorsed by the publisher.

## Supplementary Material

Supplementary Figure 1 PCA and PLS-DA scores plots of the NMR spectra of polar extracts from HD, HI and HIR tumors.

Supplementary Figure 2 Heatmap of metabolite variations characterizing tissues from MG, to HD, HI and HIR tumors.
