# Supplementary figures and images for: Metabolic Adaptations in an Endocrine-Related Breast Cancer Mouse Model Unveil Potential Markers of Tumor Response to Hormonal Therapy
Source: Front Oncol. 2022 Mar 1;12:786931. doi: 10.3389/fonc.2022.786931 (PMC8921989; doi:10.3389/fonc.2022.786931)

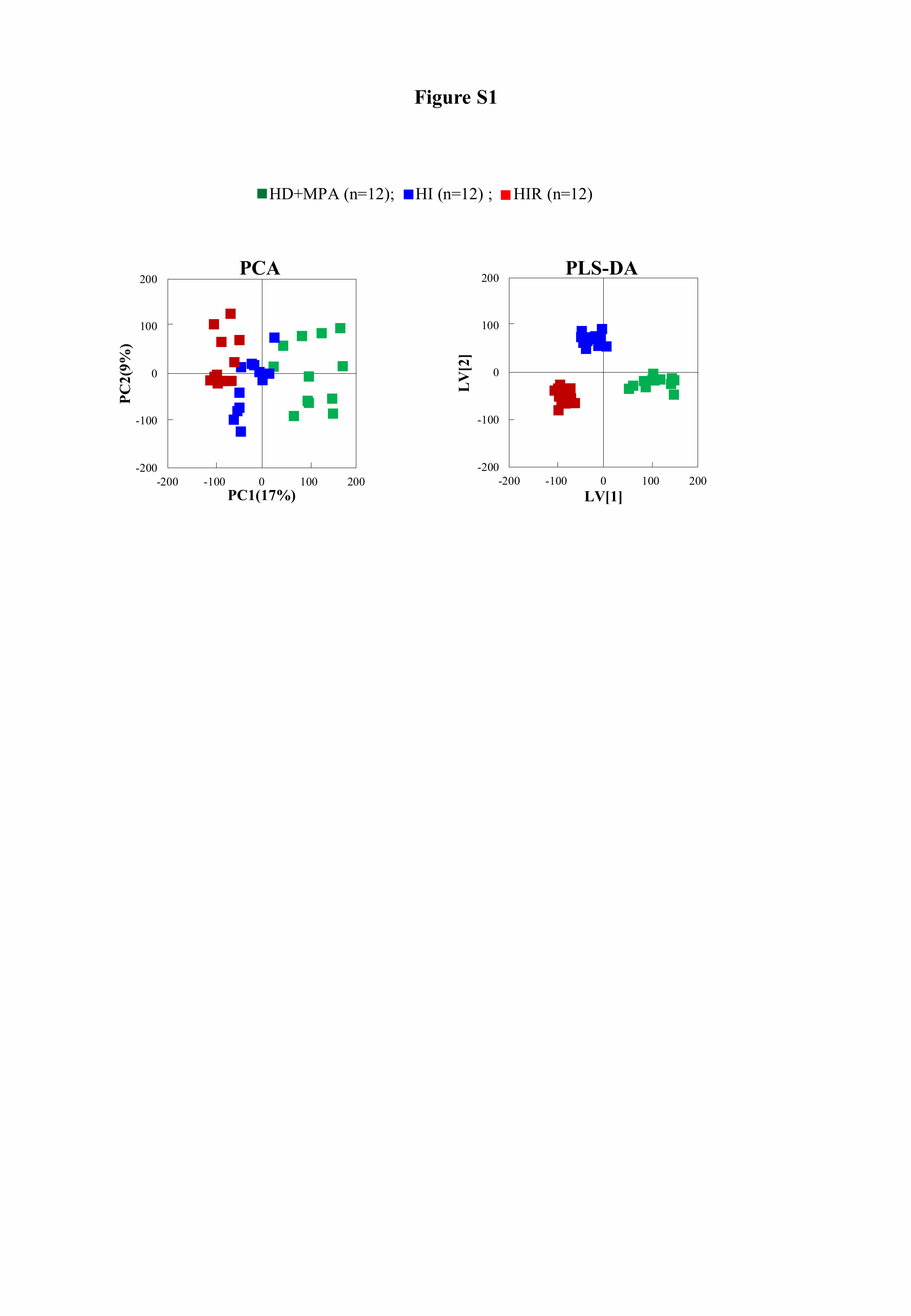

Supplement: Supplementary Figure 1 — PCA and PLS-DA scores plots of the NMR spectra of polar extracts from HD, HI and HIR tumors. [file Image_1.tif]

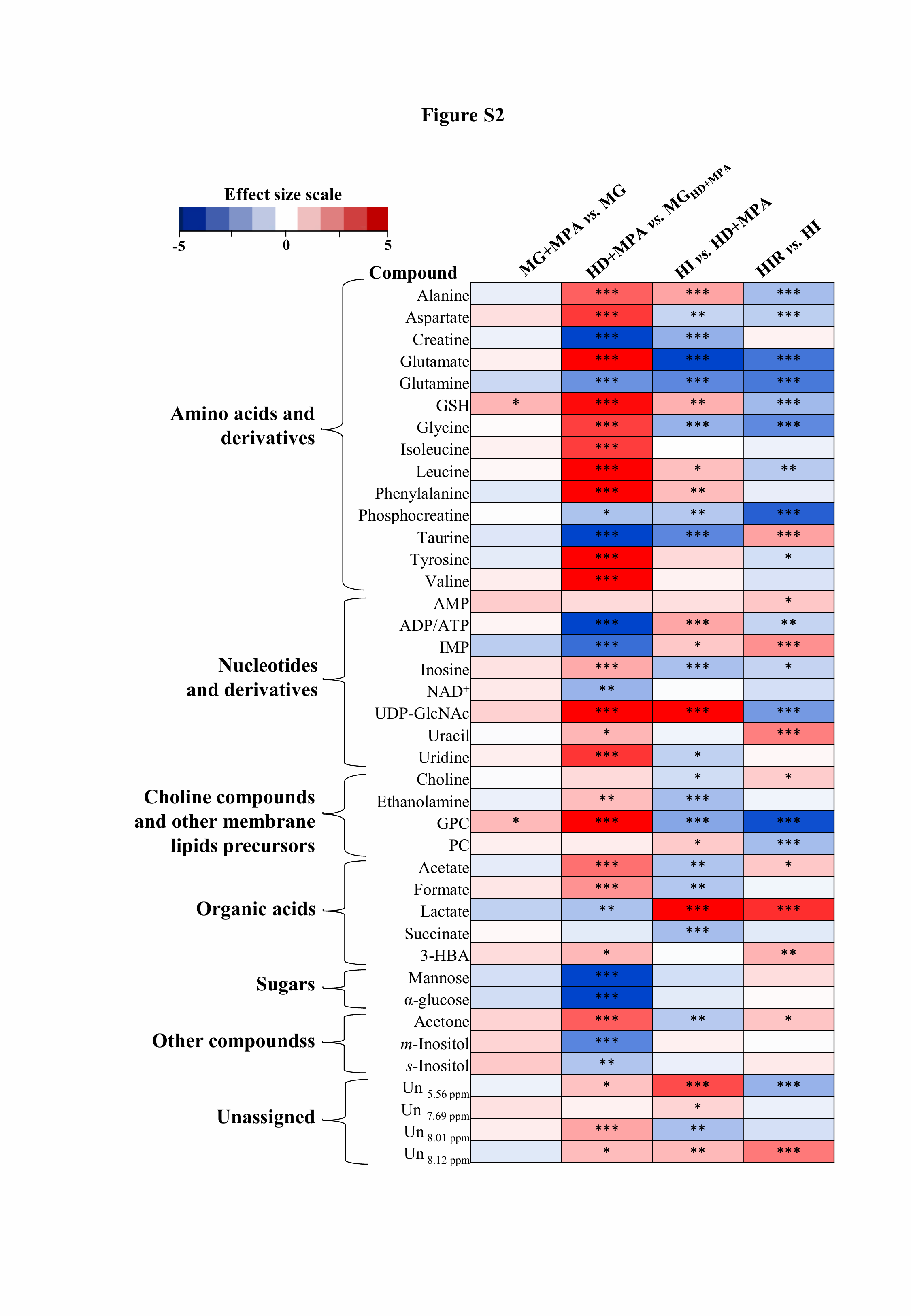

Supplement: Supplementary Figure 2 — Heatmap of metabolite variations characterizing tissues from MG, to HD, HI and HIR tumors. [file Image_2.tif]
