# Supplementary material for: Metabolic Adaptations in an Endocrine-Related Breast Cancer Mouse Model Unveil Potential Markers of Tumor Response to Hormonal Therapy
Source: Front Oncol. 2022 Mar 1;12:786931. doi: 10.3389/fonc.2022.786931 (PMC8921989; doi:10.3389/fonc.2022.786931)
Supplement: Supplementary file 3 [file Table_1.docx]

**Table S1.** List of metabolites identified in the ^1^H NMR spectra of aqueous extracts of mammary tumors (C4-HD, C4-HI and C4-HIR) and mammary gland (MG; either healthy or from tumor bearing animals). ^a^ and ^b^: metabolites identified only in mammary tumors and in the MG tissue, respectively, by NMR spectroscopy. For the majority of metabolites, found in both tissue types, the arrows in the right column indicate the relative qualitative variation between tumors and MG tissue (statistically relevant metabolite variations are listed in Table S2). The HMDB ID column indicates the metabolite identification number, as defined in reference (25). Abbreviations: 3-HBA: 3-hydroxybutyrate, ADP: adenosine diphosphate, AMP: adenosine monophosphate, ATP: adenosine triphosphate, GSH: glutathione (reduced), IMP: inosine monophosphate, NAD^+^: nicotinamide adenine dinucleotide (oxidized), UDP-GlcNAc: Uridine diphosphate *N*-acetylglucosamine. s: singlet, d: doublet, t: triplet, q: quartet, dd: double of doublets, m: multiplet.

| Compound | δ_H_ (multiplicity) | HMDB ID | Qualitative variation (tumors *vs* MG) |
| --- | --- | --- | --- |
| 3-HBA | 1.19 (d), 2.32 (dd), 2.40 (dd), 4.16 (dt) | HMDB0000011 | 🡩 |
| Acetate | 1.91 (s) | HMDB0000042 | 🡩 |
| Acetone | 2.23 (s) | HMDB0001659 | 🡩 |
| Adenosine | 3.85 (dd), 3.94 (dd), 4.30 (q), 4.43 (dd), 6.08 (d), 8.24 (s), 8.34 (s) | HMDB0000050 | 🡩 |
| ADP | 4.15 (m), 4.16 (m), 4.57 (m), 6.14 (d), 8.27 (s), 8.54 (s) | HMDB0001341 | 🡫 |
| Alanine | 1.48 (d), 3.78 (q) | HMDB0000161 | 🡩 |
| AMP | 4.01 (dd), 4.36 (dd), 4.50 (dd), 6.14 (d), 8.21 (s), 8.61 (s) | HMDB0000045 | 🡩 |
| Aspartate | 2.81 (dd), 2.67 (dd), 3.91 (dd) | HMDB0000191 | 🡩 |
| ATP | 4.21 (m), 4.29 (m), 4.39 (m), 4.50 (m), 4.61 (t), 6.15 (d), 8.27 (s), 8.54 (s) | HMDB0000538 | 🡫 |
| Choline | 3.20 (s), 3.50 (m), 4.05 (m) | HMDB0000097 | 🡩 |
| Creatine | 3.03 (s), 3.93 (s) | HMDB0000064 | 🡫 |
| Ethanol | 1.18 (t), 3.63 (q) | HMDB0000108 | 🡩 |
| Ethanolamine | 3.14 (m), 3.82 (m) | HMDB0000149 | - |
| Formate | 8.46 (s) | HMDB0000142 | 🡩 |
| Glucose | 3.23 (dd), 3.39 (m), 3.45 (m), 3.52 (dd), 3.71 (m), 3.82 (m), 3.88 (dd), 4.65 (d), 5.23 (d) | HMDB0000122 | 🡫 |
| Glutamate | 2.1 (m), 2.35 (m), 3.76 (dd) | HMDB0000148 | 🡩 |
| Glutamine | 2.14 (m), 2.45 (m), 3.78 (t) | HMDB0003423 | 🡫 |
| Glycerophosphocholine | 3.23 (s), 3.65 (m), 3.92 (m), 4.33 (m) | HMDB0000086 | 🡩 |
| Glycine | 3.56 (s) | HMDB0000123 | 🡩 |
| GSH | 2.15 (m), 2.55 (m), 2.95 (dd), 3.78 (m), 4.61 (q) | HMDB0000125 | 🡩 |
| Histidine | 3.13 (m), 3.21 (m), 3.98 (m), 7.03 (s), 7.85 (m) | HMDB0000177 | 🡫 |
| Inosine | 3.90 (dd), 4.26 (dd), 4.44, (dd), 6.10 (d), 8.18 (s), 8.35 (s)^a,b^ | HMDB0000195 | 🡩 |
| IMP | 4.02 (m), 4.36 (m), 4.50 (m), 6.15 (d), 8.23 (s), 8.58 (s) | HMDB0000175 | 🡫 |
| Isoleucine | 1.02 (d), 0.94 (t) | HMDB0000172 | 🡩 |
| Lactate | 1.33 (d), 4.11 (q) | HMDB0000190 | 🡩 |
| Leucine | 0.96 (t), 1.70 (m) | HMDB0000687 | 🡩 |
| Lysine | 1.48 (m), 1.72 (m), 1.91 (m), 3.01 (t), 3.75(t) | HMDB0003405 | 🡩 |
| Mannose^a^ | 3.37 (dd), 3.56 (t), 3.64 (m), 3.73 (m), 3.79 (m), 3.84 (m) | HMDB0000169 | 🡫 |
| Methanol | 3.35 (s) | HMDB0001875 | 🡫 |
| Methionine^b^ | 2.10 (m), 2.64 (t), 3.85 (dd) | HMDB0000696 | - |
| *m*-Inositol | 3.30 (t), 3.53 (dd), 3.63 (t) | HMDB0000211 | 🡫 |
| NAD^+^ | 4.22 (m), 4.35 (m), 4.37 (m), 4.43 (dd), 4.49 (m), 4.53 (m), 6.04 (d), 6.09 (d), 8.15 (s), 8.23 (m), 8.43 (s), 8.84 (d), 9.15 (d), 9.34 (s) | HMDB0000902 | 🡫 |
| Phenylalanine | 3.14 (m),3.30 (m), 4.00 (dd) | HMDB0000159 | 🡩 |
| Phosphocholine | 3.22 (s), 3.64 (m), 4.21 (m), 7.33 (m), 7.39 (m), 7.43 (m) | HMDB0001565 | 🡩 |
| Phosphocreatine | 3.04 (s), 3.93 (s) | HMDB0001511 | 🡫 |
| *s-*Inositol | 3.35 (s) | --- | 🡫 |
| Succinate | 2.41 (s) | HMDB0000254 | 🡩 |
| Taurine | 3.26 (t), 3.42 (t) | HMDB0000251 | 🡫 |
| Trimethylamine^a^ | 2.89 (s) | HMDB0000906 | - |
| Tyrosine | 3.06 (dd), 3.19 (dd), 3.96 (dd), 6.90 (d), 7.20 (d) | HMDB0000158 | 🡩 |
| UDP-GlcNAc | 2.08 (s), 3.55 (dd), 3.80 (m), 3.88 (dd), 3.91 (m), 3.97 (dd), 4.17 (m), 4.23 (m), 4.27 (m), 4.35 (m), 5.52 (dd), 5.98 (dd), 7.96 (d), 8.35 (d) | HMDB0000290 | 🡩 |
| Uracil | 5.80 (d), 7.54 (d) | HMDB0000300 | 🡩 |
| Uridine | 3.80 (dd), 3.91 (dd), 4.12 (m), 4.22 (dd), 4.34 (dd), 5.91 (dd), 7.88 (d) | HMDB0000296 | 🡩 |
| Valine | 0.99 (d), 1.04 (d) | HMDB0000883 | 🡩 |
