# Supplementary material for: Metabolic Adaptations in an Endocrine-Related Breast Cancer Mouse Model Unveil Potential Markers of Tumor Response to Hormonal Therapy
Source: Front Oncol. 2022 Mar 1;12:786931. doi: 10.3389/fonc.2022.786931 (PMC8921989; doi:10.3389/fonc.2022.786931)
Supplement: Supplementary file 4 [file Table_2.docx]

**Table S2.** Statistically relevant metabolite variations between all tumors (irrespective of endocrine response) and MG samples (comprising those obtained from MG, MG+MPA, MG_HD+MPA,_ MG_HI_ and MG_HIR_ groups). Positive and negative ES values correspond to metabolite increases and decreases, respectively, in tumors, compared to MG. 3-HBA: 3-hydroxybutirate, AMP: adenosine monophosphate, AXP: region arising from ATP/ADP, IMP: inosine monophosphate, GPC: glycerophosphocholine, GSH: glutathione, NAD^+^: nicotinamide adenine dinucleotide, PC: phosphocholine, UDP-GlcNAc: uridine diphosphate *N*-acetylglucosamine, Un: unassigned; * All variations remained significant upon FDR correction.

| **Compound** | **ES±ES error** | ***p-*value*** |  |
| --- | --- | --- | --- |
| **Amino acids and derivatives** | | | |
| Alanine | 2.33±0.63 | 2.20E-16 |  |
| Aspartate | 1.92±0.58 | 9.41E-14 |  |
| Creatine | -7.52±1.37 | 2.20E-16 |  |
| Glutamate | 2.33±0.63 | 2.20E-16 |  |
| Glutamine | -3.14±0.72 | 2.20E-16 |  |
| Glycine | 1.45±0.54 | 2.27E-07 |  |
| GSH | 3.66±0.79 | 9.84E-16 |  |
| Isoleucine | 4.53±0.91 | 2.20E-16 |  |
| Leucine | 6.53±1.21 | 2.20E-16 |  |
| Phenylalanine | 5.40±1.04 | 2.20E-16 |  |
| Phosphocreatine | -1.79±0.57 | 2.77E-11 |  |
| Taurine | -6.02±1.14 | 2.20E-16 |  |
| Tyrosine | 3.19±0.73 | 2.20E-16 |  |
| Valine | 6.89±1.27 | 2.20E-16 |  |
| **Nucleotides and derivatives** | | | |
| AMP | 1.90±0.58 | 2.18E-07 |  |
| ADP/ATP | -3.44±0.76 | 2.20E-16 |  |
| IMP | -2.54±0.65 | 2.20E-16 |  |
| Inosine | 0.85±0.51 | 3.19E-07 |  |
| NAD^+^ | -1.46±0.54 | 5.98E-08 |  |
| UDP-GlcNAc | 2.60±0.66 | 2.20E-16 |  |
| Uracil | 1.41±0.54 | 7.80E-09 |  |
| Uridine | 3.58±0.78 | 2.20E-16 |  |
| **Choline compounds** | | | |
| Choline | 0.71±0.50 | 2.27E-03 |  |
| GPC | 2.19±0.61 | 6.69E-15 |  |
| PC | 0.90±0.51 | 7.14E-04 |  |
| **Organic acids** | | | |
| Acetate | 1.50±0.55 | 2.11E-08 |  |
| Lactate | 1.16±0.52 | 4.79E-03 |  |
| 3-HBA | 0.73±0.50 | 2.37E-05 |  |
| **Sugars** | | | |
| Mannose | -3.57±0.78 | 2.20E-16 |  |
| α-glucose | -4.15±0.86 | 2.20E-16 |  |
| **Other compounds** | | | |
| Acetone | 1.24±0.53 | 1.27E-06 |  |
| *myo*-Inositol | -3.41±0.76 | 2.20E-16 |  |
| *scyllo-*Inositol | -1.44±0.54 | 7.83E-12 |  |
| **Unassigned resonances** | | | |
| Un _5.56 ppm_ | 1.69±0.56 | 5.68E-10 |  |
| Un _7.69 ppm_ | 0.87±0.51 | 7.80E-05 |  |
| Un _8.01 ppm_ | 0.99±0.51 | 1.03E-07 |  |
| Un _8.12 ppm_ | 1.55±0.55 | 3.45E-08 |  |
